# Atmospheric elemental carbon pollution and its regional health disparities in China

**DOI:** 10.1088/1748-9326/ad0862

**Published:** 2023-11-09

**Authors:** Yun Hang, Xia Meng, Yuzhi Xi, Danlu Zhang, Xiuran Lin, Fengchao Liang, Hezhong Tian, Tiantian Li, Tijian Wang, Junji Cao, Qingyan Fu, Sagnik Dey, Shenshen Li, Kan Huang, Haidong Kan, Xiaoming Shi, Yang Liu

**Affiliations:** 1 Gangarosa Department of Environmental Health, Rollins School of Public Health, Emory University, Atlanta, GA 30322, United States of America; 2 School of Public Health, Fudan University, Shanghai 200032, People’s Republic of China; 3 School of Public Health and Emergency Management, Southern University of Science and Technology, Shenzhen 518055, People’s Republic of China; 4 State Key Laboratory of Environmental Simulation and Pollution Control, School of Environment, Beijing Normal University, Beijing 100875, People’s Republic of China; 5 China CDC Key Laboratory of Environment and Population Health, National Institute of Environmental Health, Chinese Center for Disease Control and Prevention, Beijing 100021, People’s Republic of China; 6 School of Atmospheric Sciences, Nanjing University, Nanjing 210023, People’s Republic of China; 7 Institute of Atmospheric Physics, Chinese Academy of Sciences, Beijing 100101, People’s Republic of China; 8 State Ecologic Environmental Scientific Observation and Research Station at Dianshan Lake, Shanghai Environmental Monitoring Center, Shanghai 200235, People’s Republic of China; 9 Centre for Atmospheric Sciences, Indian Institute of Technology Delhi, Hauz Khas, New Delhi 110016, India; 10 State Key Laboratory of Remote Sensing Science, Aerospace Information Research Institute, Chinese Academy of Sciences, Beijing 100101, People’s Republic of China; 11 Shanghai Key Laboratory of Atmospheric Particle Pollution and Prevention (LAP3), Department of Environmental Science and Engineering, Fudan University, Shanghai 200433, People’s Republic of China

**Keywords:** PM_2.5_ constituents, elemental carbon, mortality burden, remote sensing

## Abstract

Previous studies have reported that atmospheric elemental carbon (EC) may pose potentially elevated toxicity when compared to total ambient fine particulate matter (PM_2.5_). However, most research on EC has been conducted in the US and Europe, whereas China experiences significantly higher EC pollution levels. Investigating the health impact of EC exposure in China presents considerable challenges due to the absence of a monitoring network to document long-term EC levels. Despite extensive studies on total PM_2.5_ in China over the past decade and a significant decrease in its concentration, changes in EC levels and the associated mortality burden remain largely unknown. In our study, we employed a combination of satellite remote sensing, available ground observations, machine learning techniques, and atmospheric big data to predict ground EC concentrations across China for the period 2005–2018, achieving a spatial resolution of 10 km. Our findings reveal that the national average annual mean EC concentration has remained relatively stable since 2005, even as total PM_2.5_ levels have substantially decreased. Furthermore, we calculated the all-cause non-accidental deaths attributed to long-term EC exposure in China using baseline mortality data and pooled mortality risk from a cohort study. This analysis unveiled significant regional disparities in the mortality burden resulting from long-term EC exposure in China. These variations can be attributed to varying levels of effectiveness in EC regulations across different regions. Specifically, our study highlights that these regulations have been effective in mitigating EC-related health risks in first-tier cities. However, in regions characterized by a high concentration of coal-power plants and industrial facilities, additional efforts are necessary to control emissions. This observation underscores the importance of tailoring environmental policies and interventions to address the specific challenges posed by varying emission sources and regional contexts.

## 1. Introduction

Ambient air pollution has been recognized as one of the leading environmental risk factors of human health with an estimated mortality burden of 8.8 million per year worldwide (Lelieveld et al 2020). In China, fine particulate matter (PM_2.5_, airborne particles with an aerodynamic diameter of less than 2.5 *μ*m) is the primary ambient air pollutant that accounted for approximately 30.8 million adult premature deaths over a 17 yr period (Liang et al 2020). In the recent decade, the total PM_2.5_ level in China has significantly decreased due to a series of air pollution control policies (Zhang et al 2019). However, PM_2.5_ consists of different chemical constituents such as elemental carbon (EC), a known human carcinogen and potentially more harmful constituent than the other major PM_2.5_ constituents (Janssen et al 2011).

Epidemiological studies have demonstrated that EC exposure is associated with a series of adverse health outcomes including deaths. However, the exact pathological mechanisms of how EC exposure leads to morbidities and mortalities remain poorly understood. Current findings suggest exposure to carbon black, which is consisting of nearly pure EC (Long et al 2013), could lead to oxidative stress and inflammation which further lead to morbidities like cancer, respiratory diseases, cardiovascular dysfunctions, and eventually deaths (Niranjan and Thakur 2017). First, it is evident that exposure to carbon black may lead to the development of cancer via DNA methylation and histone modification, which is associated with loss of cell growth control, changes in NF-kappa B, and C-fos activation (Mauderly et al 1987, 1994, Mroz et al 2008, Baccarelli et al 2009, Roller 2011). Second, carbon black exposure has been shown to result in respiratory diseases through eosinophils and mast cell infiltration (Takano et al 1998, Ben-Zimra et al 2013, Pawelczyk et al 2014). Third, EC exposure is also linked to cardiovascular disease via alteration of expression of endothelial molecules VCAM-1, ICAM-1, and TERT by triggering pulmonary cells and platelet activation (Alexeeff et al 2011, Frikke-Schmidt et al 2011, Folkmann et al 2012). In addition, EC can directly absorb solar radiation and produce a net warming effect on the regional and global climate (Bond et al 2013). Moreover, the formation, transport, and deposition of EC play an important role in the global carbon cycle that further regulates Earth’s temperature and climate (Ogren and Charlson 1983).

Considering the high PM_2.5_ level in China and the possible elevated toxicity of EC, limited ground observation-based studies reported EC pollution over several major cities (Wang et al 2019, Yin et al 2020, Luo et al 2021). Unlike developed countries such as the US which has the National PM_2.5_ Chemical Speciation Monitoring Networks (Solomon et al 2014), only a few monitoring sites in China can provide information on PM_2.5_ constituents. Several studies, based on chemical transport model simulations, have estimated large-scale black carbon (BC), a broader term that includes both pure EC and light-absorbing organic carbon compounds (Andreae and Gelencsér 2006, Chow et al 2009). These estimates are helpful for understanding PM_2.5_ in China but come with large uncertainties stemming from emission inventories (Li et al 2017, Liu et al 2022). For instance, the correlation coefficient between model simulations and observations of BC over Eastern China, where the most extensive ground measurements of PM_2.5_ constituents are available, stands at only 0.64 at the monthly level (Geng et al 2019). Furthermore, in comparison to EC, the mixed nature of BC limits its applicability in studying the sources and emissions of particulate matter. Therefore, our focus here is on EC, as it represents the pure carbon component and can aid in identifying specific combustion sources, such as diesel engines or biomass burning.

Fortunately, aerosol data retrieved by the space-borne sensor Multiangle Imaging SpectroRadiometer (MISR) onboard NASA’s Terra satellite can provide unique aerosol microphysical properties valuable for predicting PM_2.5_ constituents over regions that do not have ground monitoring sites (Liu et al 2007, Meng et al 2018a). An emerging solution to understanding the health effects of long-term EC exposure in China is combining MISR aerosol data and machine learning algorithms (Mooney and Pejaver 2018, Hang et al 2022). Here, we present a national EC model across China supported by the random forest algorithm incorporating multiple satellite datasets, atmospheric reanalysis, available ground data, and other supporting information. We then combined the model with baseline mortality data in China and pooled relative risk (RR) of EC from a published cohort study to evaluate the long-term mortality burden of EC. To our knowledge, this is the first study to estimate the long-term health impact of EC across China. Our estimates provide a great opportunity to advance the understanding of air pollution and health equity in China (Wang et al 2022). Unlike the US has well-documented the health impact of a broad list of air pollutants over different regions, few studies exist for China because the current policy designation does not contain a clear description of the concept of health equity (Wang et al 2021). A more thorough air pollutants and health monitoring framework is needed to fill the gap in assessing regional air pollution health disparities in China.

## 2. Method

### 2.1. Study design

Our study domain includes every province, autonomous region, and special administrative region in China as displayed in figure S1. We estimated EC exposure and evaluated its health impact based on modeling grid cells at a spatial resolution of 10 km. The study period is 2005–2018 that covers years with nationwide early regulations on air pollution (i.e. 11th Five-Year Plan (FYP), 2006–2010; 12th FYP, 2011–2015), the toughest-ever air quality policy (i.e. Air Pollution Prevention and Control Action Plan (APPCAP), 2013–2017), and the beginning of the most recent air policy before the break of COVID-19 (i.e. Blue Sky War, 2018–2020) (Zhang et al 2019). We selected the year 2005 as the starting point because air pollution control policies prior to 2005 were not effective (Jin et al 2016).

First, we developed a model to predict daily EC concentrations based on the random forest algorithm incorporating available ground-based EC observations, satellite data, and atmospheric reanalysis. Although there is no uniform standard to measure EC in China, we used high-quality daily EC measurements following the IMPROVE protocol (Luo et al 2021). Additionally, EC data from Hong Kong and Taiwan PM_2.5_ speciation supersites were included to improve the model’s reliability (Lin et al 2008, Huang et al 2014). Together, with the support of our collaborators in China, we were able to collect daily ground-level EC observations from 30 monitoring sites located in populous regions in China during the study period. To ensure the model’s performance, we also gathered published EC data for model validation. A summary of data sources is listed in table S1. Second, we aggregated those new predictions of daily EC to annual means and weighted by population to explore the spatiotemporal distribution of EC in China. The long-term EC trend of each provincial-level administrative region was analyzed. Finally, we combined the EC model, pooled mortality risk of EC, and baseline mortality data to assess the health impact of long-term EC exposure in China.

### 2.2. Exposure model development

A 10 km × 10 km grid was designed to cover the study domain for model development and health impact assessment. All data described below were spatially averaged or interpolated by inverse distance weighting to match modeling grid cells. The EC model was developed by integrating predictors in the random forest algorithm, a method that allows a flexible number of predictors to estimate relationships between EC concentrations and various impact factors (Breiman 2001). For example, the random forest can rank the importance of predictors by comparing normalized mean errors and *R*^2^ values between predictions before and after each predictor is permuted while others are unchanged. The out-of-bag (OOB) bootstrap sampling strategy was used to evaluate the model’s performance during our study period (Meng et al 2018b).

Considering potential natural and anthropogenic factors that can contribute to EC pollution, we utilized road length data of highway and city expressways obtained from the Institute of Geographic Sciences and Natural Resources Research of the Chinese Academy of Sciences, LandScan annual population data at 1 km resolution (https://landscan.ornl.gov/). Multiple atmospheric reanalysis datasets, such as MERRA-2 daily BC concentration simulations at 0.5° × 0.625° resolution (Randles et al 2017) was used. Because EC is a part of BC and daily EC simulations do not exist. CAMS monthly EC emissions at 0.75° × 0.75° (Inness et al 2019), GEOS-FP daily meteorological conditions (i.e. wind direction, wind speed, and planetary boundary layer height) with a spatial resolution of 0.25° × 0.3125° (0.5° × 0.625° prior to 2013) were included in the model development (Apte et al 2015). NOAA National Centers for Environmental Information Integrated Surface Dataset daily visibility data was also utilized.

Satellite-retrieved data was integrated into the EC model to ensure its capacity to capture the complicated spatiotemporal variation of EC over China. In particular, multiple aerosol data retrieved by the multi-angle MISR instrument were added to distinguish EC from other PM_2.5_ constituents (Kahn et al 2005, Chau et al 2020). Here, we used the most recent MISR level 2 version 23 aerosol data product with a spatial resolution of 4.4 km × 4.4 km (Garay et al 2020). According to the microphysical properties of EC, total AOD and calculated absorption AOD components (i.e. AOD #8 and AOD #14) were selected for the model development (Liu et al 2007). Additional satellite data of vegetation (i.e. normalized difference vegetation index (NDVI)) and cloud fraction were added to the EC model. Terra MODIS C6 monthly NDVI product (MOD13A3) at 1 km resolution was used to consider the impact of land surface type on EC concentration. Clouds and the Earth’s Radiant Energy System (CERES) product that provides the best estimate of monthly mean cloud fraction at 1° × 1° resolution was included to reduce the model’s bias contributed by cloud cover (Wielicki et al 1998).

### 2.3. Calculation of the mortality burden

We calculated all-cause non-accidental deaths over the 2005–2018 period in each province of China attributable to long-term EC exposure based on our EC model predictions, pooled RR of EC, age-specific population data in China, and the baseline mortality of health outcomes. This methodology has been previously applied to the Global Burden of Disease project and many air pollution studies in China (Apte et al 2015, Liang et al 2020). Because the mortality risk estimates of long-term EC exposure in China do not exist, we assume the toxicity of EC is constant during the study period and the RR is similar to a study published by Fischer et al (2020). All the calculations were based on the EC model’s 10 km grid cells, then aggregated to estimate the cumulative deaths in each province of China. The RR over each grid cell in each year is defined as

(1) $$\text{RR}=e^{\beta (x-x_{\text{cf}})}$$

where β represents the health effect of long-term EC exposure, equals to 0.06 (95%, CI: 0.05–0.07) (Fischer et al 2020). x is the EC concentration of each grid cell of each year. $x_{\text{cf}}$ is the counterfactual exposure level, calculated as the lowest EC level during 2005–2018. Further, we estimated the population attributable fraction (PAF) of each grid cell by:

(2) PAF=(RR−1)∕RR.

The annual absolute number of adult death (AD) counts attributable to EC of each province of each year is calculated as:

(3) AD=PAF×POP×I.

POP is the total adult population (⩾25 years old) and I is the corresponding baseline mortality rate. Annual age-specific population and all-cause mortality rate data were obtained from the National Bureau of Statistics of China (http://data.stats.gov.cn/), China Population and Employment Statistics Yearbooks, Census and Statistics Department of the Hong Kong (www.censtatd.gov.hk/), Macao (www.dsec.gov.mo/), Taiwan (www.stat.gov.tw/). Age-specific mortality data were obtained from demographic census statistics (http://data.stats.gov.cn/).

## 3. Results and discussion

### 3.1. Nationwide EC exposure model

**Figure 1.**
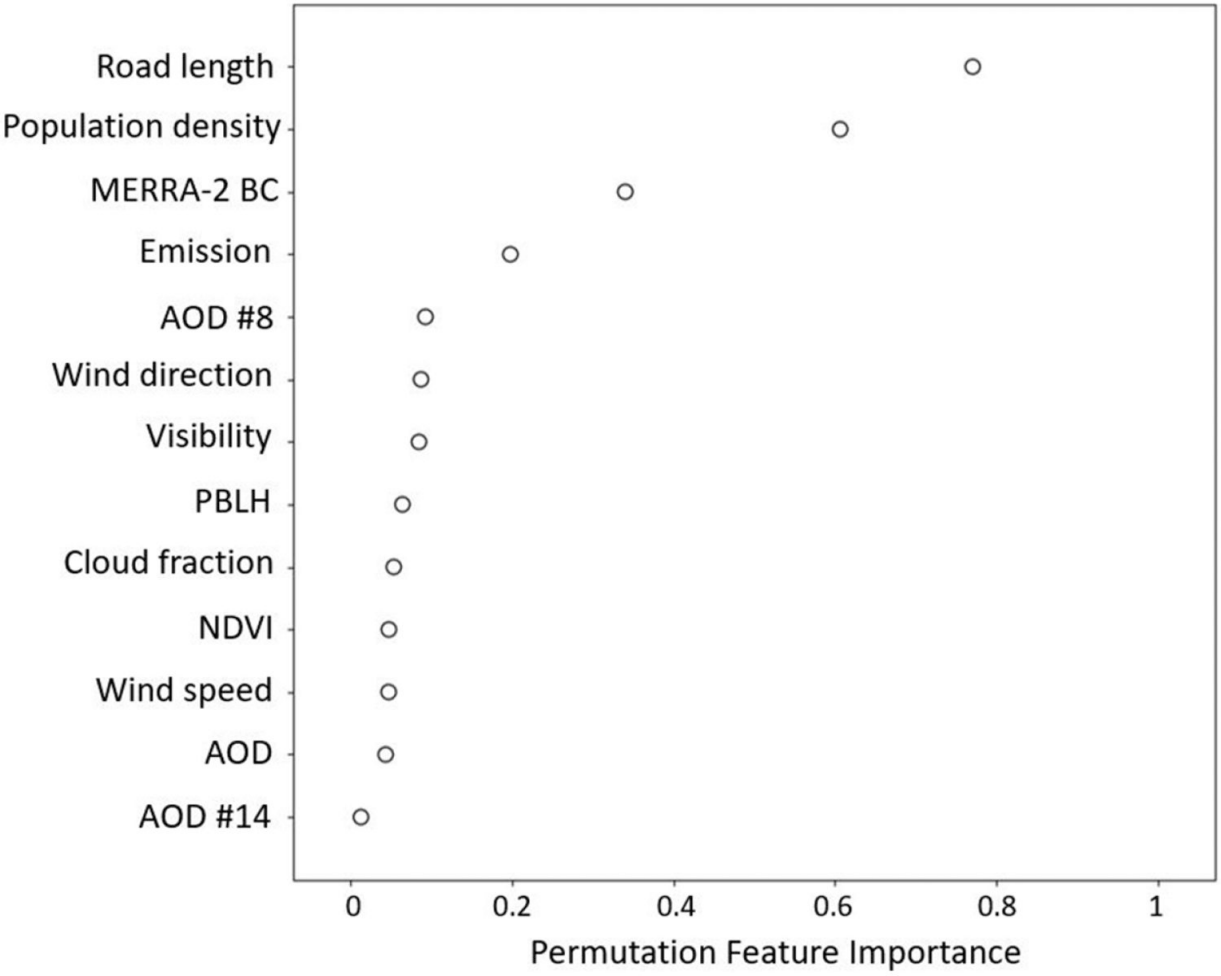
Permutation feature importance ranking of variables in the EC exposure model.

Our EC model consists of 13 predictors and five of them were observations retrieved by multiple satellite instruments (i.e. MISR AODs, MODIS NDVI, CERES cloud fraction). Figure 1 ranks model predictors by permutation feature importance. The top five predictors are road length, population density, BC concentration, EC emission, and MISR absorption AOD #8. Although the temporal resolution of MISR data is relatively low, MISR aerosol data contributes three predictors to the EC model and one of them is among the top five important predictors. It suggests that combing MISR aerosol data and machine learning algorithms to predict EC in China is feasible. Road length makes the most contribution to the EC model indicating that road traffic is a major source of EC in China, which agrees with ground-based studies at limited monitoring sites (Hou et al 2022). Regarding meteorological conditions, wind direction is the most important natural factor in the EC model. This is because air pollution levels in China are strongly modulated by mountains, hills, and rugged plateaus that make up 65% of China’s total land area. Those mountainous areas control wind directions and limit the ability of high wind speed to ventilate and disperse air pollutants. For example, the region to the east of the Taihang Mountains has frequent heavy haze pollution in winter because the mountains block the prevailing wind. Note that anthropogenic predictors such as road length and emissions contribute much more than natural predictors to this EC model, confirming that air pollution policies are of great importance to control EC pollution in China.

**Figure 2.**
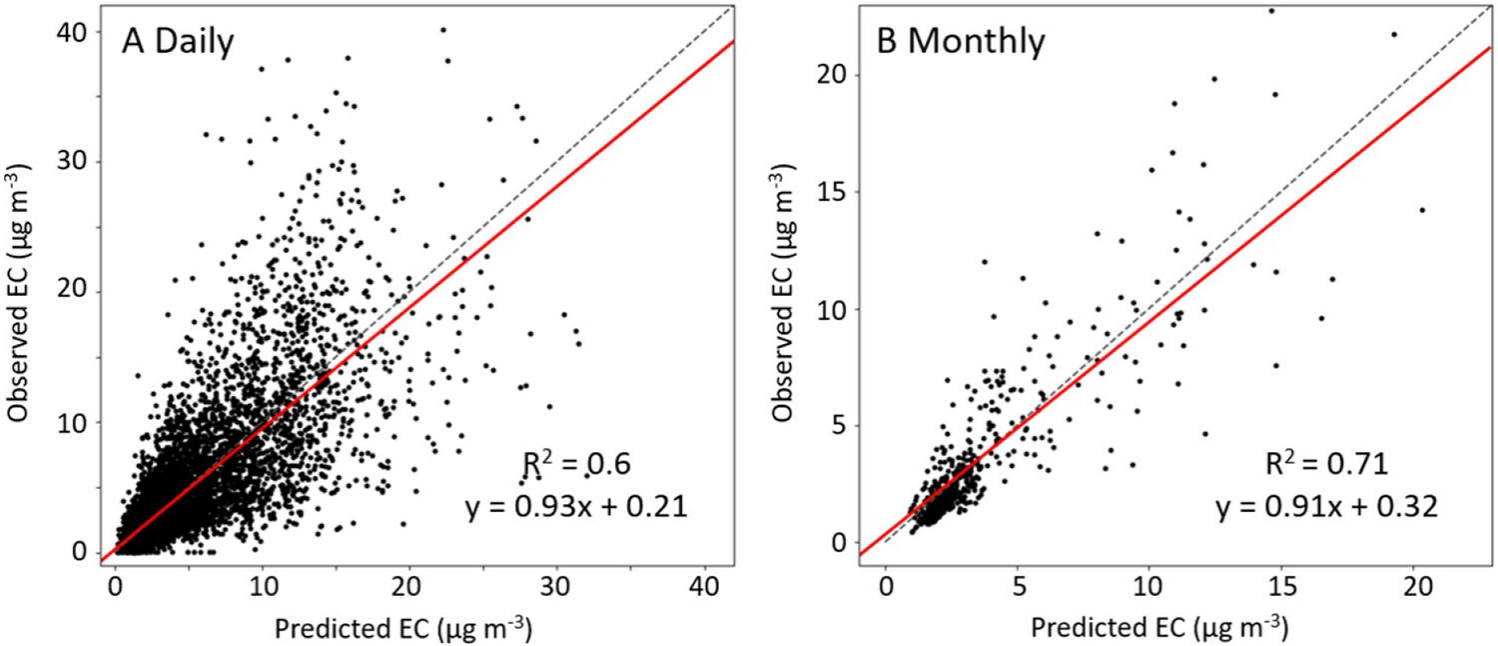
Comparison between observed and predicted EC concentrations of the newly-developed exposure model at the (A) daily and (B) monthly levels. The black dashed line is the 1:1 line. The red line is the regression line.

Figure 2 compares the linear regression and scatter plot between daily (figure 2(A)) and monthly (figure 2(B)) EC observations and predictions in China. The monthly EC calculated if more than seven daily observations were available in a month over a ground monitoring site. Compared with limited studies, our model performs with higher accuracy as the slope of regression lines between observations and predictions are close to unity, and intercepts are close to zero. The model’s OOB *R*^2^ values are 0.6 and 0.71 at daily and monthly levels. Further, we validate the EC model at seasonal levels with data collected from existing publications (table S1) to test the model’s reliability and stability (figure S2).

### 3.2. Long-term spatial variations in EC

The spatial distribution of average annual mean EC concentration in China during 2005–2018 is mapped in figure S3. In general, EC peaked at 5–6 *μ*g m^−3^ over the North China Plain (NCP), followed by the Cheng-Yu region (CY) and the Pearl River Delta (PRD) at approximately 4 *μ*g m^−3^. Low levels of EC appeared in Western China, Hainan, and Taiwan with a concentration of 1–2 *μ*g m^−3^. Compared to other PM_2.5_ constituents in China, the level of EC was much lower than the level of particulate sulfate and nitrate (Hang et al 2022, Meng et al 2023). In terms of spatial distribution, the EC pollution was mainly heavy over Hebei and Henan provinces, while nitrate and sulfate pollution was severe over many other provinces such as Shandong, Jiangsu, Sichuan, etc (Hang et al 2022, Meng et al 2023).

**Figure 3.**
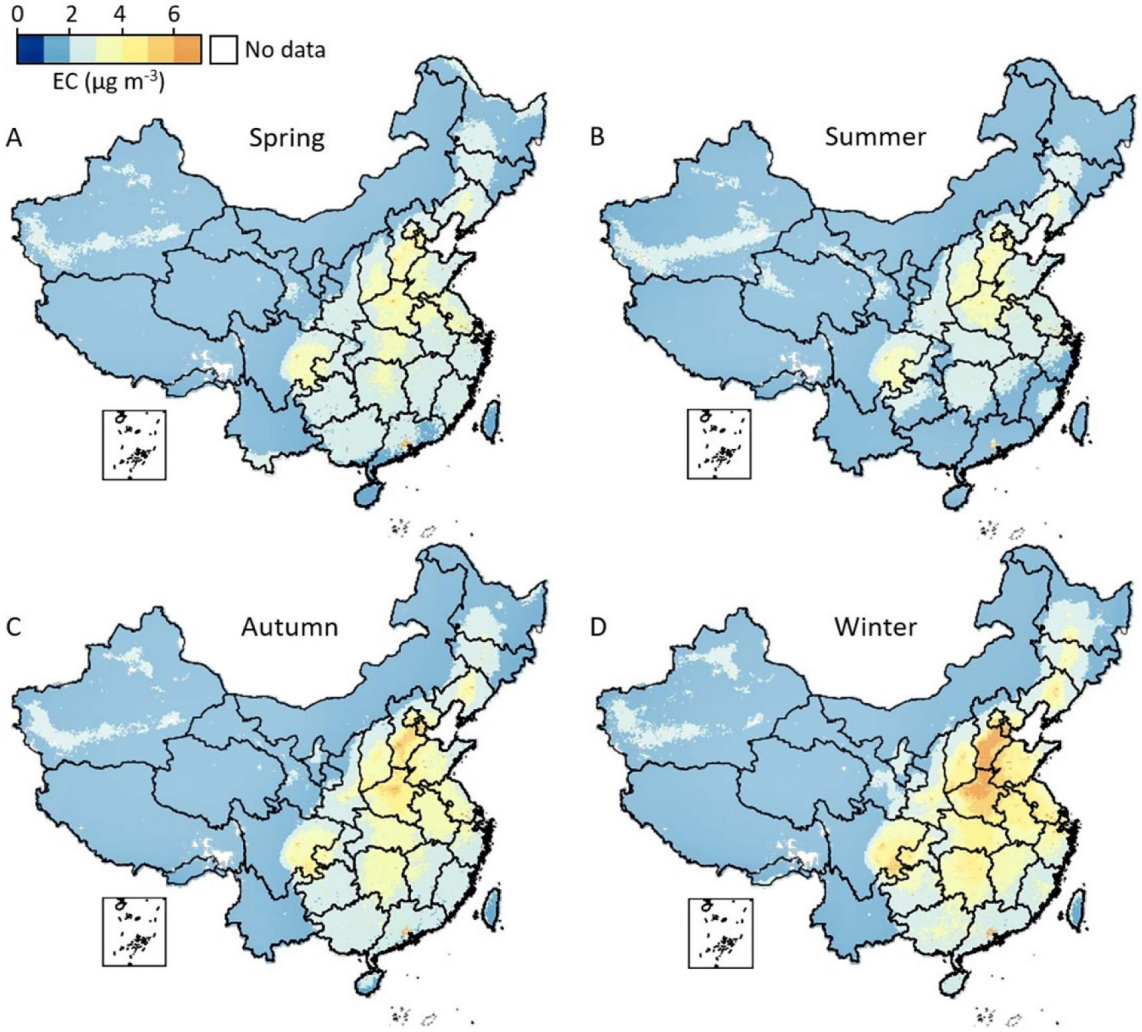
Averaged seasonal mean EC concentration in China during 2005–2018. (A) Spring (March–May), (B) summer (June–August), (C) autumn (September–November), and (D) winter (December–February).

Figure 3 compares the averaged seasonal mean EC concentration during the study period to provide more information on the potential drivers of EC pollution in China. In NCP, the highest EC level occurred in winter at 6–7 *μ*g m^−3^ due to intense coal combustion for residential heating (from 15 November to 15 March) and stable meteorological conditions (Dao et al 2021, 2022). The general EC level of NCP varied little in spring, summer, and autumn, indicating coal combustion was a main driver, despite air policies such as the APPCAP having set strict limitations on consuming coals. Interestingly, the first-tier city Beijing is located in NCP but experienced a much lower level of EC at about 4 *μ*g m^−3^, and the level was stable across the four seasons. It is likely the main driver of EC in Beijing was different from other regions in the NCP. A possible reason is that the lower EC in Beijing was contributed by the effectiveness of replacing coal with clean energy to supply heating (Luo et al 2021). Moreover, the pioneered implementation of new vehicular emissions standards in Beijing could contribute to the reduction in EC (Wang et al 2020). In addition, Beijing adopted more systematic and intensive measures for air pollution control than in its surrounding areas (Zhang et al 2016). However, the expanded civilian vehicle population and emissions were offset by reduced EC from energy consumption. The EC concentration of CY and PRD regions was relatively stable across seasons at 4–5 *μ*g m^−3^ which was contributed by traffic emissions (Zhao et al 2021). In Central China, EC was elevated to approximately 4 *μ*g m^−3^ in winter, which was influenced by the residential burning of wood or coal for heating in rural regions (Zhang et al 2014). The seasonal spatial variations of EC were mild if compared with other PM_2.5_ constituents such as nitrate which is sensitive to temperature and humidity (Hang et al 2022). By contrast, EC mainly comes from residential coal use and is more sensitive to emission changes than other PM_2.5_ constituents (Geng et al 2019).

**Figure 4.**
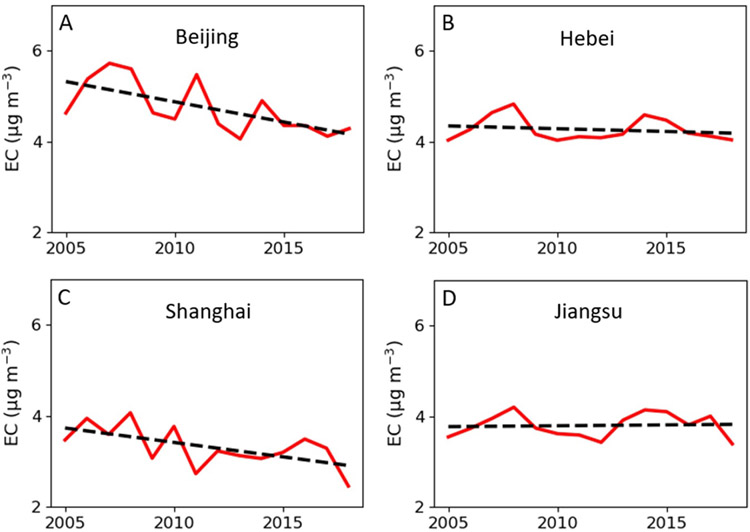
Annual mean population-weighted EC concentration (red line) of (A) Beijing, (B) Hebei, (C) Shanghai, and (D) Jiangsu. The dashed lines are calculated linear trends over 2005–2018.

We further estimated the temporal trend of annual mean EC over each province of China to explore the influence of air policy over different regions. Figure 4 compares the annual mean EC level (red line) over first-tier cities Beijing and Shanghai, and their adjacent provinces Hebei and Jiangsu. Not surprisingly, the EC concentration of Beijing and Shanghai decreased by 7.4% and 29.2% from 2005 to 2018. However, the EC level of Hebei and Jiangsu was relatively stable during the entire study period but experienced a decreasing trend after 2014. The different change of EC between first-tier cities and adjacent provinces was due to targeted air policies being initially implemented in first-tier cities and many manufacturing plants in first-tier cities were closed or moved to their adjacent provinces (Luo et al 2021). For example, strict air control policies and local vehicle regulations were implemented in Beijing in 2008 during Beijing Olympic games. Another example is the Shanghai government launched the Shanghai Clean Air Action Plan in 2013. Hebei and Jiangsu, they were benefited from nationwide air policies such as FYPs, APPCAP, and Blue Sky War to reduce EC, but the reductions were relatively small if compared with the reductions in Beijing and Shanghai. Therefore, although Hebei is located in North China and Jiangsu is in East China, their temporal trend of annual mean EC concentration exhibited a similar shape (figures 4(B) and (D)).

### 3.3. Mortality burden and regional health disparities

The mortality burden of long-term EC exposure in China has not been previously reported because predicting EC without an extensive ground monitoring network of PM_2.5_ constituents is very challenging. Another reason is the mortality risk estimates for long-term EC exposure in China do not exist. Therefore, we combined our EC exposure model, baseline mortality data of China, and pooled RR of EC from a published cohort study with 7.5 million individuals in the Netherlands (Fischer et al 2020) to estimate the all-cause non-accidental mortality attributable to EC in China.

**Figure 5.**
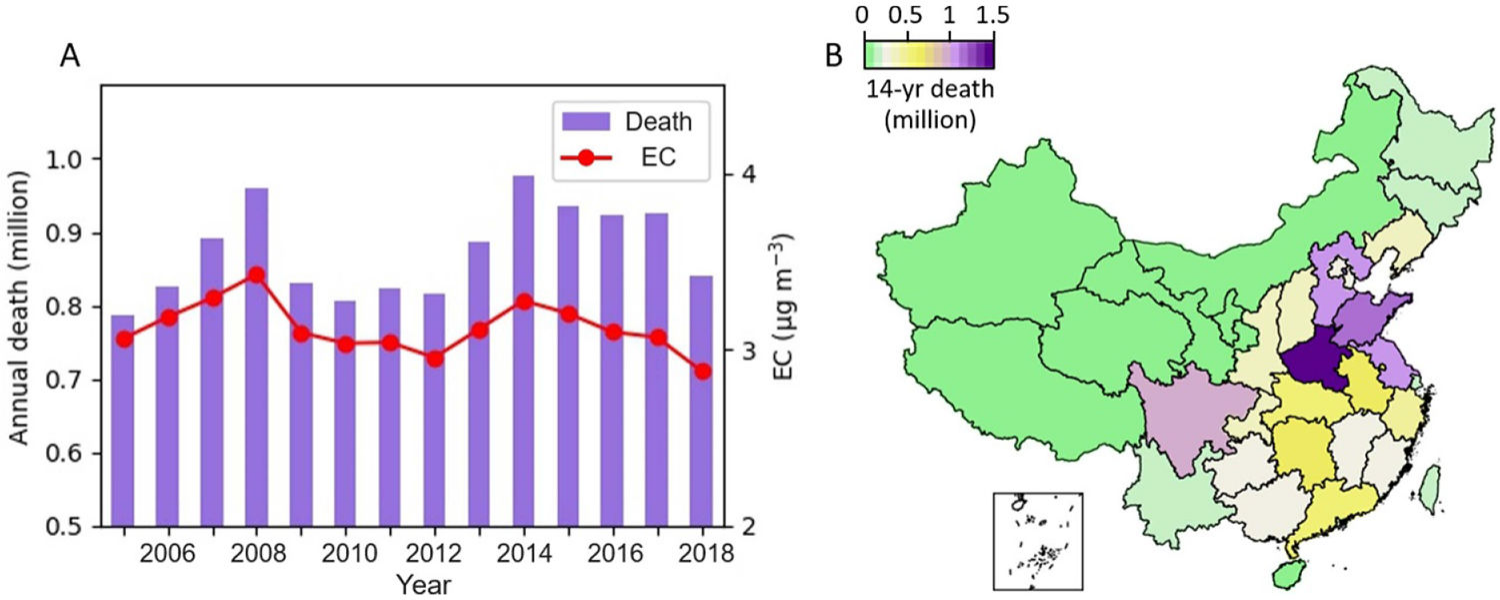
Estimated all-cause non-accidental mortality attributable to long-term EC exposure in China from 2005 to 2018. (A) Compares the national mean annual EC concentration (red dotted line, refers to the right *y*-axis) and its corresponding premature death number (purple bars, refers to the left *y*-axis). (B) Shows 14-year cumulative deaths in each province of China.

Figure 5(A) compares the national mean annual EC concentration (red line) and its corresponding annual premature death number (purple bars) from 2005 to 2018. We found that the national mean EC concentration was approximately 3 *μ*g m^−3^ with a death number ranging from 0.8 to 1 million during the study period. Although the contribution of EC to the total PM_2.5_ masses was small, the estimated mortality burden of EC was comparable to other PM_2.5_ constituents (Hang et al 2022), indicating EC is of potential disproportionally high toxicity. During the 11th FYP, the national mean EC concentration peaked at 3.4 *μ*g m^−3^ in 2008 and decreased to 3 *μ*g m^−3^ in 2010. Correspondingly, the annual death number decreased to 0.81 (95%, CI: 0.68–0.92) million. This was because the 11th FYP strengthened clean coal production and utilization, encouraged the development of coal washing and dressing. In addition, the national III vehicle standard was implemented on 1 July 2007, to control on-road vehicle emissions (Wu et al 2017). The EC level was relatively stable during the 12th FYP except a peak was observed in 2014 and resulted in an annual death number of 0.98 (95%, CI: 0.83–1.12) million. Fortunately, the Chinese government implemented the toughest ever APPCAP in 2013 and one of the main goals was to prohibit new coal-fired plants in Beijing–Tianjin–Hebei, Yangtze River Delta and PRD, and require existing coal plants to reduce emissions or be replaced with natural gas. After that, a new three-year action plan for cleaner air, Blue Sky War, was issued in June 2018 to adjust industrial structures, transform energy system toward a cleaner, more efficient energy system, and develop a green transportation system in China. As a result, the annual mean EC concentration continuously decreased after 2013 and the premature death number in 2018 was estimated as 0.84 (95%, CI: 0.71–0.96) million.

Figure 5(B) shows the estimated 14 year cumulative death number attributable to long-term EC exposure in each province of China from 2005 to 2018. Compared with the death number contributed by other PM_2.5_ constituents, the mortality attributable to long-term EC exposure in China was more localized that caused more deaths near the EC source regions. We found that the maximum of the total death number occurred in Henan province at about 1.5 million. This is because Henan is the most populated province in North China, where coal and straw were commonly used for household heating (Zi et al 2021). Following provinces with high mortality were Shandong, Hebei, Jiangsu, and Sichuan, with a cumulative death number higher than 1 million. Unlike first-tier cities Beijing and Tianjin with a death number of about 0.25 million, the mortality of Hebei was four times higher. This was partially contributed by the relocation of more polluting industry facilities from the first-tier cities to Hebei (Luo et al 2021). It has been reported that Hebei is the largest steel production province in China and production-based emissions in Hebei are much higher than in Beijing and Tianjin (Sun et al 2022). Additionally, most electricity in Hebei was generated by coal-fired plants while Beijing has been ambitious to replace coal combustion with clean energy (Liu et al 2016). Compared to Beijing, more than 20% of the population in Hebei was under 14 years old while the proportion in Beijing was approximately 10%, as reported by the National Bureau of Statistics of China in 2021. The high cumulative death number in Shandong, Jiangsu, and Sichuan was likely heavily contributed by traffic emissions from the expanding civilian vehicle population and biomass burning in rural areas. The cumulative death number in the first-tier city Shanghai was 0.1–0.2 million, which was at least five times lower than its adjacent provinces Jiangsu and Zhejiang. This reinforces that EC regulations in China were effective in first-tier cities, but need more effort to control emissions from coal-power plants, industrial facilities, and on-road vehicles in other regions, which are more vulnerable to long-term EC exposure.

## 4. Conclusions

A few limitations should be considered when interpreting the findings from our study. First, as an exposure modeling-focused study, the health burden of long-term EC exposure was calculated by pooled RR of EC in the Netherlands rather than in China, which could bring large uncertainties. The EC level of China is much higher than the level of Netherlands, and the two countries’ demographics and socioeconomic characteristics are very different. We will reassess the health risk of EC in China once a cohort study conducted in China is available. Second, limited ground-level measurements of EC might introduce uncertainties when predicting EC in areas and during the period with less or no measurements. Third, MISR has a narrow swath, which results in limited spatial coverage for the exposure estimates over eastern Tibet (figure 3). Although the missing data area exhibits an extremely low population density (figure S1), it would be beneficial to incorporate gap-filling approaches to enhance the model’s performance. We recommend future studies consider those factors to extend PM_2.5_ constituents research in China and other low- and middle-income countries (LMICs) where lack of ground monitoring networks. In conclusion, we confirm the feasibility of incorporating MISR data and machine learning to predict EC exposure in China where does not have sufficient ground data. Our study finds that the health burden of long-term EC exposure in first-tier cities was much lower than in their surrounding provinces. However, many provinces that have elevated EC concentrations are more vulnerable than people living in first-tier cities. Although air quality in China has been significantly improved, reducing health inequity among different regions may need more attention (Liu and Zhang 2019). Our findings may have important implications for future air policy development, understanding of the health effects of PM_2.5_ constituents, and protecting the health of vulnerable populations, particularly in LMICs and under-resourced regions.

## Supplementary Material

supplement

## Data Availability

All data that support the findings of this study are included within the article (and any supplementary files).
