# Supplementary material for: Atmospheric elemental carbon pollution and its regional health disparities in China
Source: Environ Res Lett. Author manuscript; Available in PMC 2024 Jul 19. (PMC11259311; doi:10.1088/1748-9326/ad0862)
Supplement: supplement [file NIHMS1957187-supplement-supplement.docx]

**Supplementary appendix**


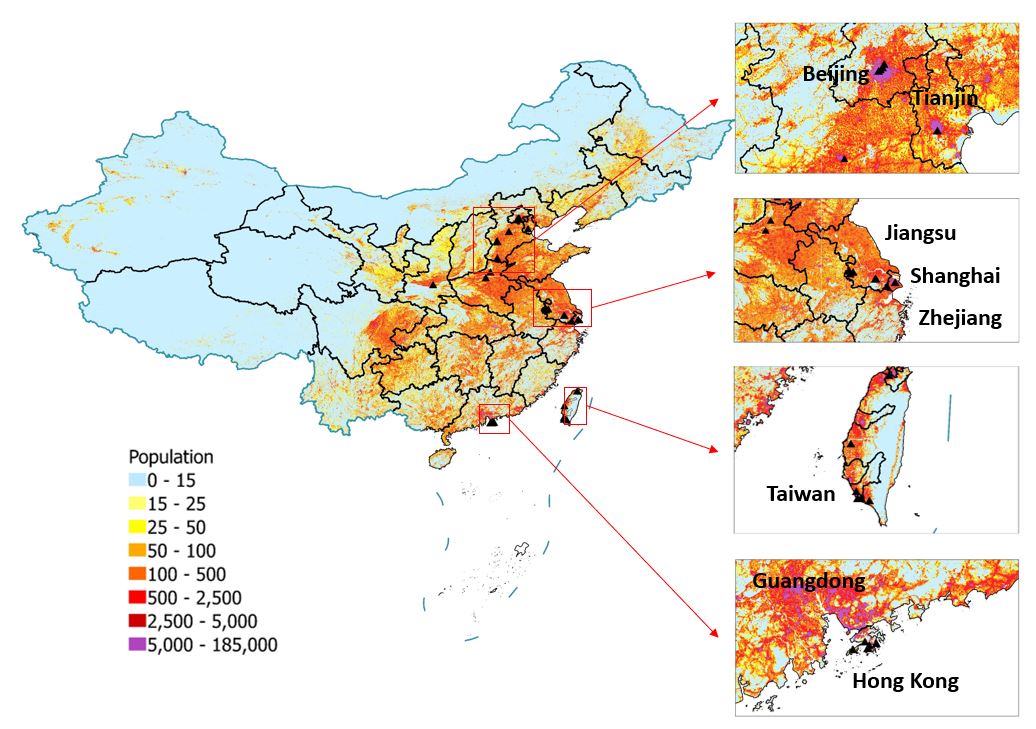


**Figure S1.** Study domain and locations of available EC monitoring sites (black triangle) in China that were included in this study. The background of this map is colored by population density (people/km^2^) derived from LandScan population data for the year 2018.


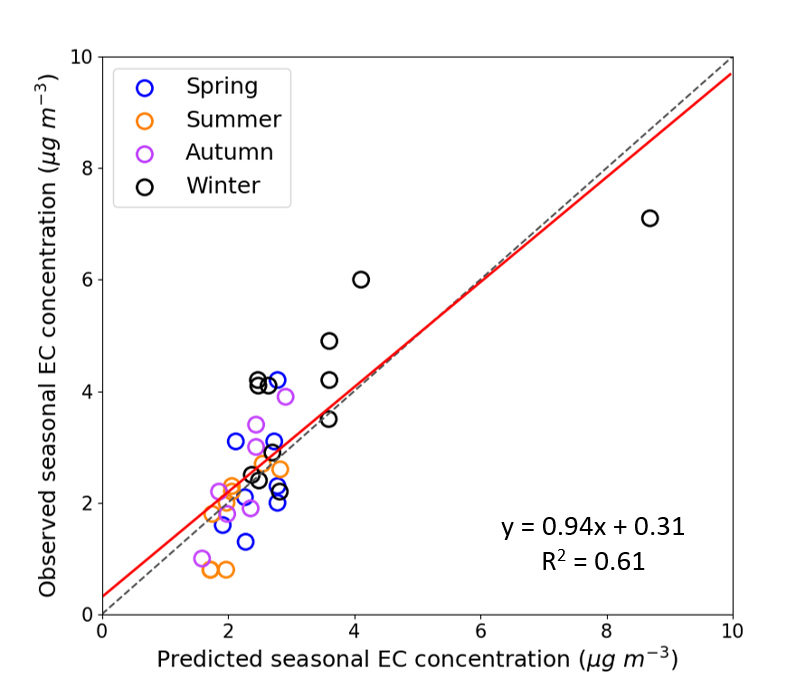


**Figure S2.** Model performance of predicting seasonal EC concentration in China. The solid red line represents the regression line between predictions and observations reposted by previous publications, and the dashed black line represents the x=y line. The regression function and R^2^ are shown at the bottom right.


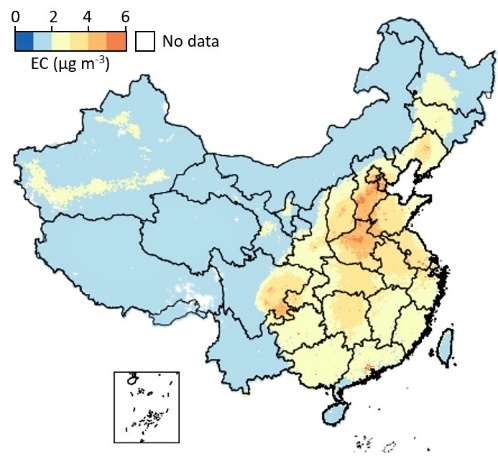


**Figure S3**. Spatial distribution of averaged annual mean EC concentration in China, 2005-2018.

**Table S1.** Summary of published EC measurements used for model validation.

| Province | Prefectural City | Period | Data Source |
| --- | --- | --- | --- |
| Jiangsu | Nanjing | 2011.3.12-31; 2011.6.10-26 | Shen et al. (2014) |
| Shanghai | Shanghai | 2009.9.1-20 | Cao et al. (2013) |
| Shanghai | Shanghai | 2013.1.5-25 | Huang et al. (2014) |
| Shanghai | Shanghai | 2013.10.16-11.14; 2013.12.21-2014.1.20; 2014.3.21-4.20; 2014.6.23-7.23 | Ming et al. (2017) |
| Zhejiang | Ningbo | 2009.7-2010.3 | Liu et al. (2013) |
| Guangdong | Guangzhou | 2004.8.16-9.17; 2005.2.1-3.8 | Duan et al. (2007) |
| Guangdong | Guangzhou | 2007.12.31-2008.1.12 | Tan et al. (2009) |
| Guangdong | Guangzhou | 2008.12-2009.2 | Yang et al. (2011) |
| Fujian | Fuzhou | 2007.4-2008.1 | Xu et al. (2012) |
| Fujian | Xiamen | 2009.4-2010.1 | Zhang et al.(2011) |
| Fujian | Xiamen | 2009.6-2010.5 | Zhang et al. (2012) |
| Hainan | Haikou | 2015.1.13-22; 2015.3.3-11; 2015.7.21-30; 2015.9.19-29 | Liu et al. (2017) |
| Hunan | Changsha | 2013.9-10; 2013.12-2014.1; 2014.4-5; 2016.7-8 | Tang et al. (2017) |

CAO, J.-J., ZHU, C.-S., TIE, X.-X., GENG, F.-H., XU, H.-M., HO, S., WANG, G.-H., HAN, Y.-M. & HO, K.-F. 2013. Characteristics and sources of carbonaceous aerosols from Shanghai, China. *Atmospheric Chemistry and Physics,* 13**,** 803-817.

DUAN, J., TAN, J., CHENG, D., BI, X., DENG, W., SHENG, G., FU, J. & WONG, M. H. 2007. Sources and characteristics of carbonaceous aerosol in two largest cities in Pearl River Delta Region, China. *Atmospheric Environment,* 41**,** 2895-2903.

HUANG, R.-J., ZHANG, Y., BOZZETTI, C., HO, K.-F., CAO, J.-J., HAN, Y., DAELLENBACH, K. R., SLOWIK, J. G., PLATT, S. M. & CANONACO, F. 2014. High secondary aerosol contribution to particulate pollution during haze events in China. *Nature,* 514**,** 218-222.

JIHUA, T., JINGCHUN, D., KEBIN, H., YONGLIANG, M., FENGKUI, D., YUAN, C. & JIAMO, F. 2009. Chemical characteristics of PM2. 5 during a typical haze episode in Guangzhou. *Journal of Environmental Sciences,* 21**,** 774-781.

LIU, B., LI, T., YANG, J., WU, J., WANG, J., GAO, J., BI, X., FENG, Y., ZHANG, Y. & YANG, H. 2017. Source apportionment and a novel approach of estimating regional contributions to ambient PM2. 5 in Haikou, China. *Environmental Pollution,* 223**,** 334-345.

LIU, D., LI, J., ZHANG, Y., XU, Y., LIU, X., DING, P., SHEN, C., CHEN, Y., TIAN, C. & ZHANG, G. 2013. The use of levoglucosan and radiocarbon for source apportionment of PM2. 5 carbonaceous aerosols at a background site in East China. *Environmental science & technology,* 47**,** 10454-10461.

MING, L., JIN, L., LI, J., FU, P., YANG, W., LIU, D., ZHANG, G., WANG, Z. & LI, X. 2017. PM2. 5 in the Yangtze River Delta, China: Chemical compositions, seasonal variations, and regional pollution events. *Environmental pollution,* 223**,** 200-212.

SHEN, G., XUE, M., YUAN, S., ZHANG, J., ZHAO, Q., LI, B., WU, H. & DING, A. 2014. Chemical compositions and reconstructed light extinction coefficients of particulate matter in a mega-city in the western Yangtze River Delta, China. *Atmospheric environment,* 83**,** 14-20.

TANG, X., CHEN, X. & TIAN, Y. 2017. Chemical composition and source apportionment of PM2. 5–A case study from one year continuous sampling in the Chang-Zhu-Tan urban agglomeration. *Atmospheric Pollution Research,* 8**,** 885-899.

XU, L., CHEN, X., CHEN, J., ZHANG, F., HE, C., ZHAO, J. & YIN, L. 2012. Seasonal variations and chemical compositions of PM2. 5 aerosol in the urban area of Fuzhou, China. *Atmospheric Research,* 104**,** 264-272.

YANG, F., TAN, J., ZHAO, Q., DU, Z., HE, K., MA, Y., DUAN, F. & CHEN, G. 2011. Characteristics of PM 2.5 speciation in representative megacities and across China. *Atmospheric Chemistry and Physics,* 11**,** 5207-5219.

ZHANG, F., XU, L., CHEN, J., YU, Y., NIU, Z. & YIN, L. 2012. Chemical compositions and extinction coefficients of PM2. 5 in peri-urban of Xiamen, China, during June 2009–May 2010. *Atmospheric Research,* 106**,** 150-158.

ZHANG, F., ZHAO, J., CHEN, J., XU, Y. & XU, L. 2011. Pollution characteristics of organic and elemental carbon in PM2. 5 in Xiamen, China. *Journal of Environmental Sciences,* 23**,** 1342-1349.

**References**
